# Treg: A Promising Immunotherapeutic Target in Oral Diseases

**DOI:** 10.3389/fimmu.2021.667862

**Published:** 2021-06-10

**Authors:** Yujing Zhang, Jihua Guo, Rong Jia

**Affiliations:** ^1^ The State Key Laboratory Breeding Base of Basic Science of Stomatology (Hubei-MOST) & Key Laboratory of Oral Biomedicine Ministry of Education, School & Hospital of Stomatology, Wuhan University, Wuhan, China; ^2^ Department of Endodontics, School & Hospital of Stomatology, Wuhan University, Wuhan, China

**Keywords:** Treg, immunotherapy, oral diseases, periodontitis, oral cancer

## Abstract

With the pandemic of COVID-19, maintenance of oral health has increasingly become the main challenge of global health. Various common oral diseases, such as periodontitis and oral cancer, are closely associated with immune disorders in the oral mucosa. Regulatory T cells (Treg) are essential for maintaining self-tolerance and immunosuppression. During the process of periodontitis and apical periodontitis, two typical chronic immune-inflammatory diseases, Treg contributes to maintain host immune homeostasis and minimize tissue damage. In contrast, in the development of oral precancerous lesions and oral cancer, Treg is expected to be depleted or down-regulated to enhance the anti-tumor immune response. Therefore, a deeper understanding of the distribution, function, and regulatory mechanisms of Treg cells may provide a prospect for the immunotherapy of oral diseases. In this review, we summarize the distribution and multiple roles of Treg in different oral diseases and discuss the possible mechanisms involved in Treg cell regulation, hope to provide a reference for future Treg-targeted immunotherapy in the treatment of oral diseases.

## Introduction

Regulatory T cell (Treg) was first reported to be involved in maintaining self-tolerance as early as the 1970s, but there was still a lack of specific molecular markers (1). Until 1995, Sakaguchi et al. found that the IL-2 receptor α-chain (CD25) was constitutively expressed on Treg cells, the concept of regulatory T cells was formally put forward (2). Treg, as a subset of CD4^+^ T lymphocytes, is crucial for maintaining self-tolerance and immune homeostasis. It characteristically expresses the transcription factor forkhead box P3 (FOXP3), identified as the main regulator for Treg development and function (3).

During the emergency of COVID-19, maintenance of oral health has increasingly become the main challenge of global health due to the possibilities of increasing viral transmission (4). Oral diseases are ones of the common public health problems. Among them, periodontitis is the most important cause of adult permanent teeth loss; lip and oral cancer, as the 15th most common cancer worldwide, is closely related to the quality of human life (5). As the epitome of the whole body system, the oral cavity is affected by a variety of diseases and disorders, including apical periodontitis and periodontitis as acute and chronic infectious diseases, autoimmune diseases such as Pemphigus Vulgaris (PV), oral cancer, and oral potentially malignant disorders (6). Increasing evidence shows that the regulation of Treg cell number and function in different diseases have opposite expectations. In autoimmune diseases, Treg cells are expected to be more stable and polyclonal and play a practical immunosuppressive role (7). On the other hand, Treg cells suppress the anti-tumor immune response, accelerate tumor proliferation and metastasis in some tumors. Therefore, Treg targeted immunotherapy is often at the forefront of anti-tumor therapy (8). However, it is worth noting that the research on the role and regulatory mechanisms of Treg cells in oral diseases is incomplete. In this review, we discuss the immunosuppressive mechanisms of FOXP3^+^ Treg cells and summarize their distribution and function in different types of oral diseases, especially the possible mechanisms involved in the regulation of Treg distribution, proliferation, and function, to provide some new prospects that may eventually apply to clinical treatment.

## Treg Biology

Treg cells are involved in maintaining immune tolerance, accounting for 5% - 10% of CD4^+^ T cells in the peripheral circulation (2, 9, 10). According to their different origins, the Treg population is further divided into three subgroups: thymus-derived Treg cells (tTregs), peripheral Treg cells (pTregs) and induced Treg cells (iTregs). tTregs are mainly derived from T-cell precursors stimulated by TCR signal and costimulatory molecules in the thymus; while pTregs are induced from naïve CD4^+^ T cells that are exposed to cytokine TGF-β and IL-2 in the periphery. Besides, iTregs are induced in the TGF-β environment *in vitro* with unstable Treg phenotypes (11). The transcription factor forkhead box P3 (FOXP3) has been considered as a specific Treg molecular marker, essential for its differentiation, phenotype maintenance, and immunosuppressive function (12). FOXP3 gene mutation leads to the impairment of Treg cells development and inhibition function, resulting in human IPEX (immune dysregulation, polyendocrinopathy, enteropathy, X-linked syndrome) disease and scurfy in rodents, respectively (13, 14). IPEX is also named X-linked autoimmunity allergic dysregulation syndrome (XLAAD). Patients with the disease will present many immunopathological symptoms within infancy, including enteropathy, diabetes, dermatitis, thyroid disease, and anemia (15). The scurfy mice are characterized by scaly and ruffled skin, spleen and lymph node enlargement, and premature death about a few weeks after birth (16, 17).

The intrinsic commitment and stable maintenance of the Treg lineage depend on the sustained high expression of FOXP3. It endows Treg with a variety of essential characteristics, including high expression of CD25 and cell surface molecules like cytotoxic T-lymphocyte–associated antigen 4 (CTLA-4), suppression of proinflammatory cytokines as IL-4 and IL-17 conversely (18). At the same time, FOXP3 can interact with ∼700 target genes and multiple microRNAs to regulate the development and function of Treg collectively (19, 20).

In addition, the stability of the Treg lineage is also regulated by epigenetics. The FOXP3 locus contains several conserved noncoding enhancer sequences (CNS) that are targeted by epigenetic modifications and several transcription factors (21). Mothers against decapentaplegic homologue 3 (SMAD3) and nuclear factor of activated T (NFAT) bind to CNS1 after the activation of TGF-β signal and promote FOXP3 expression, which plays a key role in the induction of pTreg cells (22). During tTreg cell development, CpG elements within CNS2 manifest demethylation progressively. Besides, both runt-related transcription factor 1 (RUNX1) and core-binding factor subunit (CBF-β), forming a trimeric complex at the CNS2, enable the stable expression of FOXP3 (23). CNS3 also facilitates FOXP3 transcription *via* the combination of c-Rel (in the NF-κB pathway) after TCR signal activation (24). In general, Treg stability is closely related to the complex and interrelated genetic landscape shaped by FOXP3 and the higher-level epigenetic regulation involved in the induction and maintenance of FOXP3 expression.

However, the stability of Treg cells is not always immutable. It has strong adaptability in an inflammatory environment. Under the local inflammatory stimuli, dendritic-cell-derived IL-6 can induce Treg cells to transform into Th17 cells (25, 26). Th17 cells, as the representative of CD4^+^ T cell pro-inflammatory subsets, mainly secrete pro-inflammatory cytokine interleukin IL-17 (27). Retinoid-related orphan receptor γt (ROR γt) is a unique lineage-specific transcription factor of Th17 (28). Both Th17 and Treg cells share a common key regulatory factor TGF-β, which participates in the activation of RORγt and FOXP3 (29). In the stimulation of proinflammatory cytokines such as IL-6 or IL-21, a low concentration of TGF-β induces the development of Th17 cells, correspondingly, a high concentration of TGF-β can promote the differentiation of naive CD4^+^ T cells into Tregs and maintain immune tolerance (30). IL-6 and IL-21 also upregulate the expression of RORγt *via* inhibiting FOXP3 activity in a signal transducer and activator of transcription 3 (STAT3) dependent manner (31). In addition, pro-inflammatory cytokines tumor necrosis factor-α (TNF-α) could down-regulate the expression of FOXP3 by binding with tumor necrosis factor receptor RII (TNFRII) and interfere with the inhibitory function of Treg cells (32). At the same time, it promotes the recruitment of protein kinase C-θ (PKC-θ) and inhibited Treg function by activation downstream Akt signal (33). Therefore, the inflammatory microenvironment may induce the instability of Treg cells, and further exacerbate inflammatory responses and tissue damage in inflammatory diseases, such as apical periodontitis.

On the contrary, tumor-infiltrating Treg cells showed quite active inhibitory phenotypes, with high expression of immune checkpoint molecules, including CTLA-4, programmed cell death 1 (PD-1), T cell immunoglobulin and mucin domain-containing protein 3 (TIM-3), lymphocyte activation gene-3 (LAG-3) and T-cell immunoreceptor with Ig and ITIM domains (TIGIT) (34). Tumor cells can suppress the secretion of IL-6 in dendritic cells by the overexpression of indoleamine 2,3-dioxygenase (IDO), inhibit the reprogramming of Treg cells to Th17 cells, and further enhance the stability of Treg cells in the tumor microenvironment by silencing the expression of the Akt/mTOR pathway (35). Therefore, the enhanced stability of Treg cells in the tumor microenvironment may contribute to the inhibition of anti-tumor immunity and immune escape.

## Mechanisms of Treg-Mediated Suppression

Treg cells exert immunosuppressive function through cell-contact-independent or cell-contact-dependent mechanisms. Cell-contact-independent mechanisms mainly include secretion of inhibitory cytokines and metabolic disruption. Cell-contact-dependent mechanisms mainly include modulation of antigen-presenting cell (APC) function and mediating cytolysis or apoptosis of target cells.

### Induction of Inhibitory Cytokines

Treg cells secrete cytokines with vital immunosuppressive function, including IL-10, TGF-β, and IL-35 (36). IL-10 downregulates the expression of class II major histocompatibility complex (MHC II) and costimulatory molecules, and directly inhibits the synthesis and secretion of inflammatory factors, thus inhibiting the capacity of antigen-presenting cells (APCs) and playing an anti-inflammatory role (37). Interestingly, IL-10-producing T regulatory type 1 (Tr1) cells are also endowed with similar inhibitory functions without FOXP3 expression (38). TGF-β also affects the differentiation, development, and function of various immune cells. TGF-β inhibits APCs’ function and limits cytotoxic T lymphocyte (CTL) proliferation (39). At the same time, immature CD4^+^ T cells could be induced to Tregs by antigen stimulation in an enriched TGF-β environment *in vitro* (40). Stimulated Treg cells also exert an immunosuppressive effect in the form of cell-cell interaction with persistently expressing TGF-β at a high level on the cell surface (41). As a novel member of the IL-12 family, IL-35 is another inhibitory cytokine explicitly secreted by Treg cells, involved in the maintenance of its maximum inhibitory function. Ectopic expression of IL-35 confers regulatory activity on naive T cells in a titrable fashion, whereas recombinant IL-35 alone is sufficient to suppress T-cell proliferation (42).

### Regulation of Antigen-Presenting Cell (APC) Function

CTLA-4, constitutively expressed in Treg cells, is an inhibitory receptor associated with the T cell costimulatory molecule CD28 (43). CTLA-4 and CD28 compete for costimulatory receptors (CD80, CD86) on antigen-presenting cells, resulting in the downregulation of these two costimulatory molecules, thus inhibiting the T cell response (44, 45). Furthermore, CTLA-4 promotes the upregulation of the enzyme IDO by dendritic cells (DCs), which catalyzes the decomposition of tryptophan, an essential amino acid. The potential downstream effects lead to cell cycle arrest and more sensitivity to apoptosis of effector T cells, along with impairment of APCs function (46). Besides, lymphocyte activation gene 3 (LAG-3/CD223) is highly expressed on the surface of Treg cells, which combines with MHC class II molecules in higher affinity than CD4. It inhibits DC function and immunostimulatory capacity through the inhibitory signal pathway mediated by immunoreceptor tyrosine-based inhibition motif (ITAM) (47). Blocking LAG-3 attenuates the inhibitory effect of Treg cells, while the ectopic expression of LAG-3 endows CD4^+^ T cells the inhibitory activity (48).

### Mediating Cytolysis or Apoptosis of Target Cells

Treg cells also cause immunosuppression by inducing target cell death *via* cell contact. Treg cells kill target effector cells, which are mediated by releasing granzymes A and B in the perforin dependent or independent manner (49–51). Additionally, Tumor-necrosis-factor-related apoptosis-inducing ligand-death receptor 5 (TRAIL-DR5) pathway has been proved to be an important component of Treg-induced cytotoxicity (52).

### Disruption of Metabolic Pathways

Another potential mechanism of Treg-mediated suppression is the metabolic blockade. Treg cells highly express the high-affinity IL-2 receptor (CD25), resulting in competitive consumption of IL-2 with effector T cells. Therefore, the effector T cells are prone to Bim-mediated apoptosis for the deprivation of the crucial metabolic and survival cytokines (10, 53). Treg cells express the ectoenzymes CD39 and CD73, which hydrolyze adenosine triphosphate (ATP) or adenosine diphosphate (ADP) to cAMP and adenosine, driving the accumulation of adenosine nucleosides and disrupting effector cell metabolism (54). Treg cells also promote the transfer of inhibitory second messenger cAMP to an effector T cell *via* cell contact-dependent gap junction and unexpectedly inhibit the immune function of effector T cell (55).

## Distribution and Functions of Treg Cells in Oral Diseases

### Treg Cells in Apical Periodontitis

Apical periodontitis is a local inflammatory immune response caused by bacterial infection in root canals, which often leads to periapical tissue damage and alveolar bone destruction (56). Thus, the balance between the host pro-inflammatory and anti-inflammatory responses supposedly determines the progression and outcome of apical periodontitis, which is regulated by different types of CD4^+^ T helper cells, including at least Th1, Th2, Th17, and Treg cells (57). As a potential protective subset of CD4^+^ T cells, accumulating studies have revealed that the beneficial role of Treg cells in restricting the overactivity of the periapical inflammatory response (58, 59).

The number of Treg cells was found remained at relatively low levels from days 7 to 21 (acute phase, the lesions markedly expanded in 3-dimensional directions) after induced periapical lesions of the lower first molars in rats, and then increased significantly by day 35 (chronic phase, the lesions expanded slowly). Interestingly, the ratio of IL17^+^/Foxp3^+^ and the number of osteoclasts correlated negatively with Treg cells (60). In addition to artificially induced acute periapical lesions in animal models, some human studies on chronic periapical lesions assessed the expression of FOXP3, which was associated with the histological type of lesion, the intensity of the inflammatory infiltrate, and the thickness of the cystic epithelial lining. Chronic periapical lesions include periapical granulomas (PGs), radicular cysts (RCs), and residual radicular cysts (RRCs). Periapical granulomas are the most common type of chronic apical periodontitis. It is granulomatous tissue composed of lymphocytes, fibroblasts, and other inflammatory cells to replace the normal bone structure. With the persistence of chronic inflammation, the epithelial cells of Malassez are stimulated by cytokines and growth factors, proliferate into epithelial masses, then liquefy and necrose in the center, and gradually form into RCs (61). The RRCs are defined as radicular cysts which remain in the jaw without proper treatment after the affected tooth was extracted (62). FOXP3 expression in RRCs, RCs, and PGs increased sequentially. The number of FOXP3^+^ cells was significantly higher in the inflammatory infiltrate grade III, followed by that in grades II and I (63).

In another study, the percentage of Foxp3^+^ Treg cells continued to increase after pulp exposure and was negatively correlated with (sphingosine-1-phosphate receptor 1) S1P1-positive cells by day 14 after the induction of periapical lesions in rats. Upregulated S1P1 triggers a series of intracellular responses to promote the receptor activator of nuclear factor kappa B ligand (RANKL) expression, which is related to osteoclast formation during the pathogenesis of periapical bone destruction (64). Besides, S1P1 promotes inflammatory cell infiltration and inhibits the function of Treg through the Akt-mTOR pathway in the acute stage. Therefore, the complex and precise regulatory network between S1P1 signal and Treg cells better explains the process of periapical lesions.

By contrast, inhibition of Treg function with anti-GITR (a phenotypic marker of Treg cells) in mice impelled the exacerbation of severity of periapical lesions at 14 and 21 days, increased expression of pro-inflammatory cytokines and destructive tissue mediators, thus preventing the formation of the inactive/stable status. Similar results were observed in CCR4KO mice. Conversely, the expansion of Treg cells attenuates lesion progression *via* the injection of cytokine C-C motif ligand 22 (CCL22)-releasing particles in the root canal system in a CCR4-dependent manner (58). These findings suggest that Treg chemoattractant application may be a promising option in the treatment of apical periodontitis.

A recent trial is also yielding promising results that Treg cells were enriched around regenerating tissues in the root canals of dogs after regenerative endodontic treatment (RET). In vitro, stem cells from the apical papilla (SCAP) promoted the conversion of pro-inflammatory T cells to Treg cells. It may suggest that the anti-inflammatory and anti-apoptotic abilities of upregulated Treg cells promote successful tissue repair and regeneration *via* releasing more cytokines and pro-healing growth factors, which may create an appropriate immune microenvironment for tissue regeneration (59).

These findings highlight that the infiltration of Treg cells is crucial for preventing the progression of apical periodontitis and promoting tissue regeneration. Treg cell dynamics plastically regulate pathogenic Th1, Th2, or Th17 cell phenotypes to maintain normal homeostasis and restrict inflammatory reaction’s overactivity (65). Therefore, promoting endogenous Treg recruitment-based therapy may provide a promising strategy for the treatment of periapical lesions and osteolytic diseases. At the same time, Treg enrichment creates an appropriate immune microenvironment for tissue regeneration, which lays a biological foundation for regenerative endodontic treatment. In the future, the researches on the effectiveness and biosafety of chemokine controlled release system and the exact role of Treg in the regeneration process will be conducive to the theoretical basis into clinical reality.

### Treg Cells in Periodontitis

Most tissue damage in periodontitis is caused by the host immune response to infection, although the accumulation of plaque microorganisms is the initiating factor (66). Therefore, controlling the host immune-inflammatory response remains a challenge for periodontitis therapeutically interventions. Different clinical studies have described Treg accumulation preferentially in infected tissues, limiting the immune response. For instance, a large number of Treg cells has been reported in middle and advanced chronic periodontitis biopsies than gingivitis (67). Moreover, other studies have shown that chemokines such as CCL17 and CCL22 are more abundant in tissues with higher inflammatory infiltration, which seems to recruit more Treg cells from inflammatory sites in a CCR4 dependent manner (68). However, some FOXP3^+^ cells may function differently from conventional Treg cells. A small population of IL-17A^+^FOXP3^+^ cells were found in periodontitis, but not in gingivitis, suggesting the functional plasticity of Treg cells transforming into inflammatory Th17 cells in the periodontitis environment (69).

On the other hand, the defect of Treg cells function is identified in many animal models to promote the progression of periodontitis. In the *A. actinomycetemcomitans* induced mice model of periodontitis, inhibiting the function of Treg cells by anti-GITR resulted in alveolar bone resorption and increased inflammatory cell infiltration, accompanied by the decrease of IL-10, TGF-β, and CTLA-4 (70). A similar phenomenon was observed in the IDO knockout mouse model along with lipopolysaccharide (LPS)-induced inflammation, as IDO affects the metabolism of Treg cells (71). In an experimental periodontitis model, the phenotype and function of Treg cells were also affected. The down-regulated Foxp3 expression and the damage of the inhibitory effect of Treg cells on osteoclast differentiation further promoted Th17-driven bone loss. The hypermethylation of CpG sites in the Foxp3 locus caused by periodontitis may be responsible for its function impairment (72). In a recent study, the possible reason for the aggravation of periodontal disease during pregnancy has been attributed to Treg cells’ shortage. The expression of Foxp3, TGF-β, CTLA-4, and CD28 in the gingiva of pregnant mice was reduced after periodontal disease induction. Simultaneously, the frequency and inhibitory ability of Treg cells in cervical lymph nodes were also down-regulated *in vitro* test, with the increase of inflammatory Th17 cells (73).

Currently, gratifying achievements have been reported in biochemical recruitment and positive regulation of Treg cells. Local or systemic administration of IL-35 also retards alveolar bone resorption in periodontitis mice *via* regulating the balance of Th17/Treg, down-regulating RANKL, and inducing osteoprotegerin (OPG) production (74). Similarly, an injectable and biomolecule-delivery of poly(L-lactic acid) (PLLA) nanofibrous spongy microspheres (NF-SMS) promote Treg enrichment, amplification, and Treg-mediated immunotherapy against bone loss in a mouse model of periodontitis *via* significantly releasing miRNA and IL-2/TGF-β (75). Exosomes from periodontal ligament stem cells, as communication mediators, are also involved in the regulation of Treg cell distribution and play an essential role in immunomodulation. Compared with normal conditions, the exosomes of periodontal ligament stem cells isolated from *Porphyromonas gingivalis* lipopolysaccharide (LPS) induced periodontitis environment transfer less miR-155-5p and increased Sirtuin-1 (SIRT1) protein into CD4^+^ T cells, and then led to the up-regulation of Th17 and the relaxation of Tregs, thus exacerbating the inflammatory microenvironment of periodontitis (76). In addition, the ratio of Th17/Treg also inclines by oral administration of all-trans retinoic acid (ATRA) in experimental periodontitis and thus providing protection against periodontitis (77).

Therefore, these findings indicate that Th17/Treg ratio imbalance is considered a critical role in the procession of periodontitis. Treg cells suppress immunopathology to avoid extensive periodontal tissue damage. It has been proved to suppress osteoclast differentiation through cell-cell contact way by Treg cells (78). Inhibitory cytokines released by Treg cells, such as IL-10 and IL-4, are also largely involved in the inhibition of RANKL expression (79). On the contrary, Th17 induces the maturation of osteoclasts by promoting the expression of RANKL, accelerating the resorption and destruction of alveolar bone (80). Therefore, Treg cells and Th17 cells are considered the key cells to connect the immune system and bone. Existing studies have shown that periodontitis is closely related to diabetes (81), rheumatoid arthritis, cardiovascular diseases (82), and other systemic diseases (83). However, at present, most of the clinical treatment methods for periodontitis are still the basic treatment for its initiating factors. Therefore, exploring new immunotherapy for periodontitis in humans may provide potential help for the macro-control of systemic diseases. In the future, more researches are needed to understand the diversity and plasticity of Treg subsets for a more advanced and safer drug delivery system.

### Treg Cells in Head and Neck Squamous Cell Carcinoma

Immune escape is a characteristic of head and neck squamous cell carcinoma (HNSCC) (84). Treg cells might contribute to the occurrence and progression of HNSCC by suppressing antitumor immunity (85). Multiple pieces of evidence have described that the number and inhibitory activity of Treg cells is enhanced in tumors and peripheral circulation of patients with HNSCC, compared with healthy donors, along with the upregulated CD39, CD62L, CTLA-4, and FOXP3 (86–89). In addition, Treg level was proved to have a significant linear and positive correlation with tumor grades (90). Another study showed that the percentage of Treg in peripheral blood lymphocytes was also increasing correspondingly with the tumor malignant degree and lymph node metastasis. The higher the malignancy, the more activated Treg subsets (91). In the process of oral precancerous lesions to oral squamous cell carcinoma, Treg accumulation has also been widely proved, with the increase of the degree of epithelial dysplasia (92). Treg cells undoubtedly play a hostile role in the development of HNSCC. In precancerous lesions, the inflammatory response is at the peak, which is mainly maintained by Th17 cells with high levels of inflammatory cytokines, such as IL-2, IL-6, and IL-17. However, as the disease progresses, the increased level of TGF-β released by cancer cells promotes Treg differentiation, downregulates Th17 cells, further accelerating tumor progression (93).

However, there are some conflicting results about the prognostic value of Treg cells in HNSCC. Several data sustained that the high frequency of Treg cells in primary lesions and lymphogenic metastases were associated with a poor prognosis (94, 95). In contrast, other studies described that high Treg infiltration was associated with better overall survival (OS) of HNSCC (96, 97). These apparent contradictions were further explained in a recent study. Echarti et al. studied the effect of Treg cells on overall survival (OS) under different immune phenotypes and found that higher Treg cells level tended to worsen OS in “immune desert (stromal cytotoxic T cells (CTL) were ≤50 cells/mm^2^)” and “immune excluded” tumors, but in “inflamed (intraepithelial CTL were >500 cells/mm^2^)” tumors, high Treg cells significantly improved OS. This indicates that the prognostic value of Treg depends mostly on the inflammatory state of the tumor (98).

Another cross-sectional study showed that the amount of Treg cells increased and persisted in HNSCC patients after adjuvant chemoradiotherapy (CRT) compared with untreated or surgery-only patients and were resistant to activation-induced cell death (AICD) or cisplatin *in vitro*. These Treg cells have a stronger inhibitory function after CRT, which may be related to the upregulated latency-associated peptide (LAP), the glycoprotein A repetitions predominant (GARP), and CD39. This may be a potential driving factor for Immunotherapy resistance and relapse of HNSCC (99).

These studies suggest that Treg cells can block the effectiveness of antitumor immunity and contribute to tumor immune escape. Therefore, the reasonable strategy of depleting Treg cells or weakening their inhibitory functions should be pursued for immunotherapy (100). At present, blocking Treg-related immune checkpoint receptors (ICR) through immune checkpoint inhibitors (ICIS) has become one of the most promising strategies for anti-cancer therapy, such as new monoclonal antibodies against CTLA-4, programmed cell death-ligand 1(PD-L1), and PD-1 (101). Although ICIS has been approved for clinical application, the compensatory mechanisms in the tumor microenvironment, such as the up-regulation of other immunosuppressive molecules, remain as potential challenges in cancer treatment (102). Therefore, the study of combined therapy strategy for ICIS targeted Treg cells may bring hope to optimize the anti-tumor immunotherapy (34).

### Treg Cells in Oral Mucosal Diseases

Oral mucosa is a vital barrier tissue to protect the oral cavity from the invasion of pathogens and foreign antigens. It was found that FOXP3^+^ Treg cells were highly abundant in oral mucosa than in secondary lymphoid tissues and other mucosal barrier sites, and they expressed a large number of CTLA-4 and tissue retention molecule CD103. This indicates that a uniquely large number of highly active Treg cells are needed to maintain oral mucosal immune quiescence. Interestingly, Treg cells in oral mucosa were mainly dependent on the recruitment and migration of exogenous Treg cells, rather than in-situ induction (103). Abnormal numbers of Treg cells caused many types of oral mucosal diseases. For example, patients with autoimmune disease Pemphigus Vulgaris (PV) showed a decreased frequency of Treg cells, but an increased number of Th17 cells, with the reduced expression of CCL22 (104). In patients with chronic inflammatory disease aphthous ulcer, the frequency of Treg cells in peripheral blood and their inhibitory function were both down-regulated, which may be related to the decreased expression of IDO (105). In contrast, the number of Treg cells increased in precancerous lesions of oral mucosal tissues. Through the comparative study of oral epithelial precursor lesions (OEPL) and oral squamous cell carcinoma (OSCC), the expression of CD25 and FOXP3 was found to be positively correlated with the malignant degree of oral epithelial lesions (92). In the precancerous condition of oral lichen planus (OLP) and precancerous lesion actinic cheilitis (AC), FOXP3^+^ cell infiltration increased, and CD8^+^/FOXP3^+^ cell ratio decreased, suggesting the promoting role of Treg in cancer progression (106, 107). In addition, during the progress from premalignant lesions to cancer, Th1 and Th17 phenotypes gradually inclined to Treg phenotype in spleen and lymph nodes (108). Therefore, Treg cells play an irreplaceable part in maintaining the immune homeostasis of the oral mucosal barrier. However, anti-Treg immunotherapy may contribute to slow down the progression of precancerous lesions. In addition, inducing the conversion of Treg to Th17-like phenotype may provide a potential prospect for intervention the progress of precancerous lesions.

**Figure 1:**
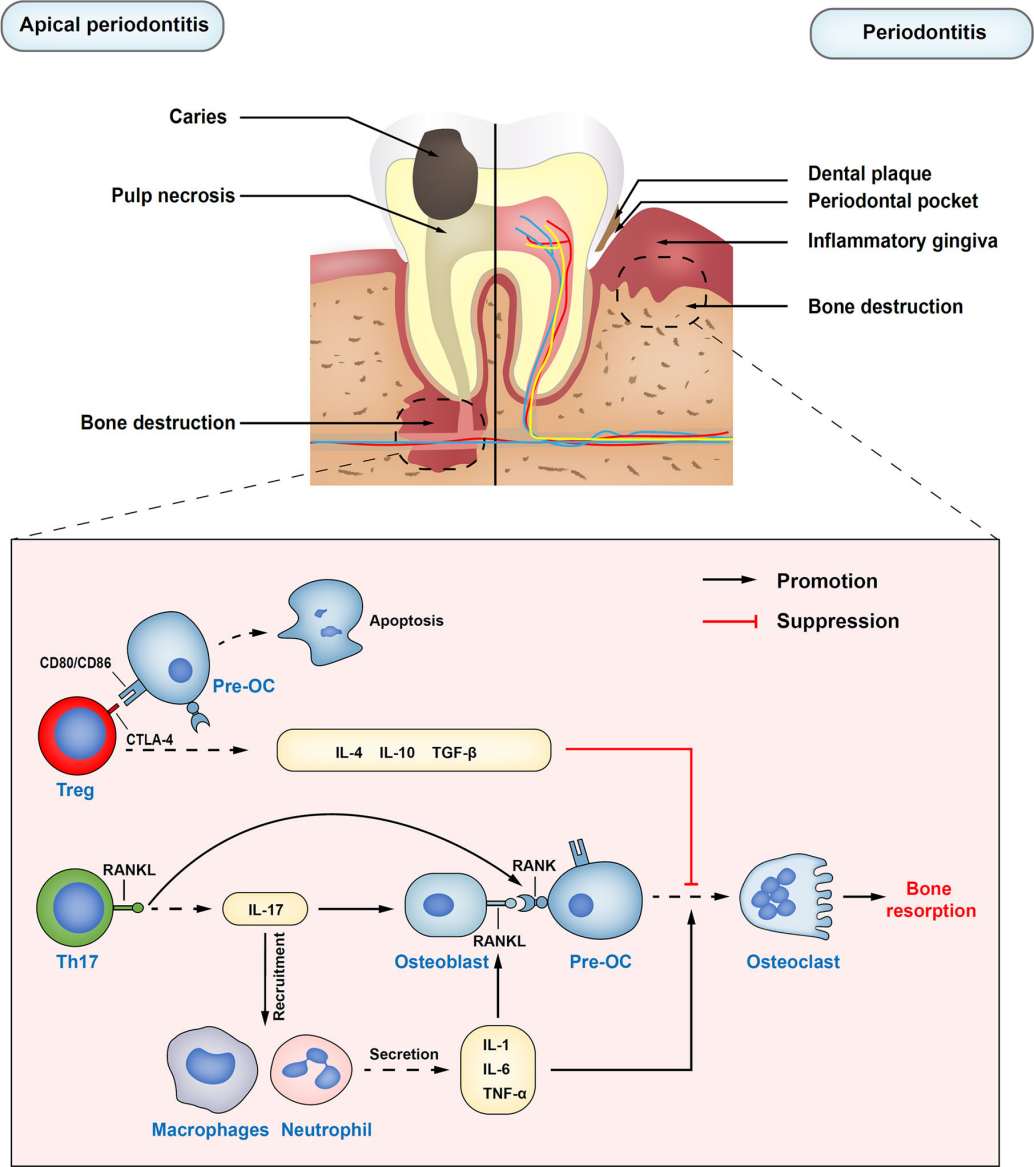
Immune regulatory functions of Treg cells in apical periodontitis and periodontitis. Treg cells inhibit the differentiation of osteoclast precursors into osteoclasts by secreting inhibitory cytokines, such as IL-10, IL-4, and TNF-β. Also, inhibiting receptor cytotoxic T-lymphocyte–associated antigen 4 (CTLA-4) on Treg cells directly in contact with osteoclast precursors costimulatory molecules CD80 and CD86, which can induce the production of indoleamine 2,3-dioxygenase (IDO) and induce the apoptosis of osteoclast precursors. Th17 cells upregulate osteoblasts and self-expressed receptor activator of nuclear factor kappa B ligand (RANKL) through the release of inflammatory cytokine IL-17. At the same time, IL-17 plays an important role in the mobilization and recruitment of immune cells, stimulating the release of local inflammatory factors, resulting in the expansion of osteoclasts, and the aggravation of inflammatory response. Pre-OC, osteoclast precursors; RANK, the receptor activator of nuclear factor kappa B.

**Figure 2:**
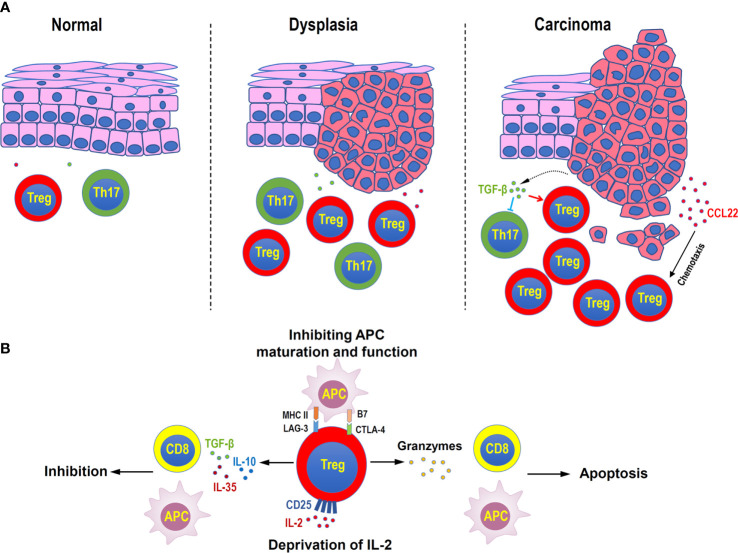
Distribution and functions of Treg cells in head and neck squamous cell carcinoma. **(A)** Treg cells increase during the disease progression in oral mucosal dysplasia and squamous cell carcinoma. Malignant cells can secret CCL22 to attract Treg cells, or secret TGF-β to suppress inflammatory Th17 cells. **(B)** Immune suppressive mechanisms of Treg cells in head and neck squamous cell carcinoma. Treg cells can secret inhibitory cytokines, such as TGF-β, IL-10, and IL-35, to suppress the functions of antigen-presenting cells (APC) and CD8^+^ effector T cells, directly kill effector or APC by granzymes, consume of IL-2 by highly expressing CD25, and negatively regulate the maturation and functions of APC by immune checkpoint molecules, such as LAG-3 and CTLA4.

In summary, Treg cells function like a double-edged sword, which plays a protective role in inhibiting the course of inflammatory diseases such as apical periodontitis and periodontitis (**Figure 1**), and autoimmune diseases, but accelerate the deterioration of precancerous lesions in oral mucosa and HNSCC (**Figure 2**).

## Regulatory Mechanisms of Treg Cells Recruitment, Proliferation, and Function in Oral Diseases

**Figure 3:**
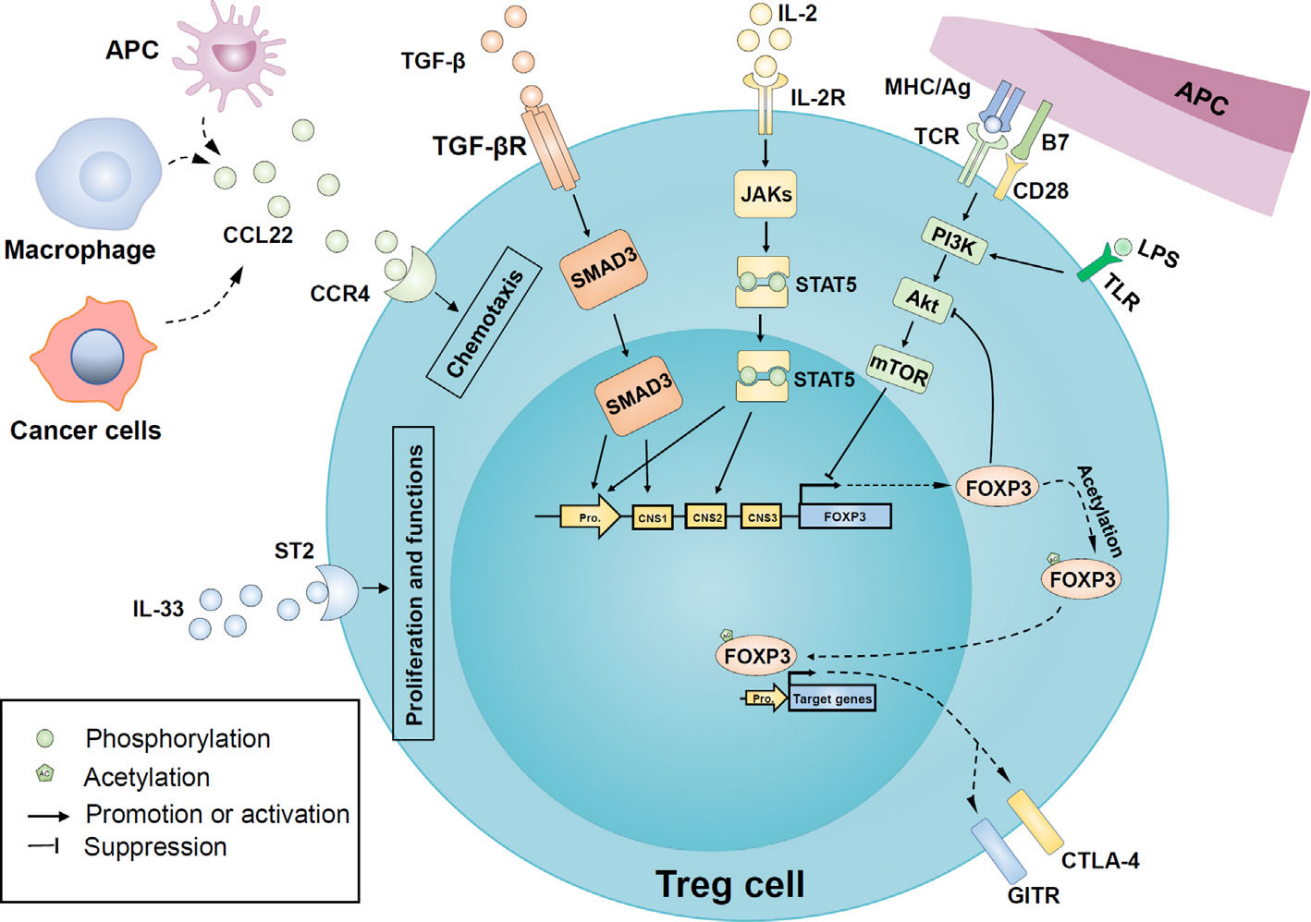
Regulatory mechanisms of Treg cells recruitment, proliferation, and function in oral diseases. Macrophages and antigen-presenting cells upregulate the release of cytokine C-C motif ligand 22 (CCL22) in an inflammatory environment and recruit more Treg cells to local tissues in a CC-chemokine receptor 4 (CCR4) dependent manner. Similarly, tumor cells are involved in the release of CCL22 in head and neck cancer. In addition, the binding of IL-2 and its receptor CD25 activated the JAK/STAT5 signaling pathway to induce FOXP3 expression. Transforming growth factor β (TGF-β) also has a positive effect on FOXP3 expression by activating mothers against decapentaplegic homologue 3 (SMAD3) transcription factors. Besides, IL-33 binds to the IL-1 receptor-like 1 (ST2) and further promotes the expression of FOXP3 and proliferation of Treg cells. The PI3K-Akt-mTOR pathway activated by inflammatory Toll-like receptor (TLR) or T cell receptor (TCR) signals may be involved in the FOXP3 expression inhibition and the regulation of Treg proliferation, while FOXP3 can negatively feedback on Akt activation. Post-translational modification of mature FOXP3 protein, such as acetylation, enhances both stability and activity of FOXP3. FOXP3 can endow Treg with typical characteristics, such as cytotoxic T-lymphocyte–associated antigen 4 (CTLA-4) and glucocorticoid-induced tumor necrosis factor receptor family-related protein (GITR). APC, antigen-presenting cells; CNS, conserved non-coding sequence; IL-2R, IL-2 receptor; LPS, lipopolysaccharide; mTOR, mechanistic target of rapamycin; PI3K, phosphoinositide 3-kinase; JAKs, Janus kinases; STAT5, signal transducer and activator of transcription 5; PI3K, phosphoinositide 3-kinase; mTOR, mammalian target of rapamycin; TGF-βR, TGFβ receptor; TLR, Toll-like receptor.

The recruitment, proliferation, and function of Treg cells are regulated by various complex regulatory networks, including cytokines, intracellular signaling pathways, epigenetic modification, and post-translational modification. These regulatory pathways affect the stability and plasticity of Treg from the cellular level to the expression of crucial genes (**Figure 3**).

### CCL22-CCR4 Axis

Many pieces of evidence suggest that the CCL22-CCR4 axis is related to the regulation of Treg cells, involving different types of oral diseases. CCL22 was originally recognized as a chemokine produced by dendritic cells and macrophages under the stimulation of bacterial components. It induces the migration of target cells by binding to its specific receptor C-C chemokine receptor type 4 (CCR4) (109). CCR4 is specifically expressed on human Treg cells in response to its chemotaxis (110). In the chronic inflammatory environment, CCL20 expression is upregulated by the proinflammatory cytokine IL-1β, which is further enhanced by the TGF-β-SMADs pathway through an enhancer upstream of the CCL20 promoter (111). NF-κB is a significant mediator of inflammation. Activated NF-κB can transactivate CCL22 expression. CCL22 also can activate NF-κB, forming a positive feedback loop (112). Interestingly, CCR4 expression is also upregulated by NF-κB activation mediated by TNF-α (113), highlighting the essential roles of NF-κB in the CCL22-CCR4 axis.

In apical periodontitis, studies have shown that CCL22 combined with CCR4 seems to be able to recruit more Treg cells into periapical lesions of mice. CCR4 depletion significantly impaired the migration ability of Tregs and increased the severity of periapical lesions, associated with the expansion of pro-inflammatory cells and tissue destruction factors. On the contrary, local administration of CCL22 in wild-type (WT) mice attenuated periapical lesions with increased Treg number, but failed in CCR4KO mice, suggesting that CCL22 promotes Treg cell migration in a CCR4 dependent manner (58). Besides, it has been reported that LPS promotes the secretion of CCL22 in macrophages by downregulating the expression of miR-34a in the apical periodontitis model of rats. The high expression of CCL22 is parallel to the frequency of Foxp3^+^ Treg cells (114).

In periodontitis, chronic periodontitis patients showed high levels of CCL22 and CCR4 compared with healthy donors (68). It was early observed in murine and canine experimental periodontitis that the release of CCL22 particles could recruit more Treg cells to inflammatory sites, and significantly reduce the alveolar bone resorption (115). Furthermore, in experimental periodontitis, CCR4KO mice and the blockade of CCL22 in WT mice both showed impairment of Treg migration, accompanied by the expansive osteoclastogenic cytokine and increased inflammatory bone loss. Adoptive transfer of CCR4^+^ Treg cells to the CCR4KO mice or exogenous release CCL22 provided by poly (lactic-co-glycolic acid) (PLGA) microparticles rescued the increased disease phenotype by promoting migration of Treg cells (116).

Similar regulatory axes have also been described in oral cancer. CCL22, as an oncogene, is upregulated in oral cancer specimens to promote the migration and infiltration of Treg cells. Silencing CCL22 expression showed opposite effects. CCL22 expression in oral cancer cells was induced by IL-1β secreted by cancer-associated fibroblasts, suggesting a new therapeutic prospect by targeting the IL-1β-CCL22-CCR4 signaling axis for the treatment of oral cancer (117). Moreover, CCL22 is the target of tumor suppressor miR-34a. In cancers, CCL22 is unregulated by the suppression of miR-34a mediated by TGF-β (118).

Therefore, these findings suggest that the CCL22-CCR4 axis is involved in Treg recruitment in a variety of oral diseases, and the diverse regulation of the CCL22-CCR4 axis according to treatment goals may provide a potential immunotherapeutic target.

### The Roles of Interleukins

A variety of interleukins participate in the stability of Treg phenotype and inhibitory function through different mechanisms. Through the stable expression of FOXP3 and CD25, IL-2 is irreplaceable for the development, stability, and function of Treg cells (119). Multiple studies have shown that IL-2 receptor signaling can mediate the phosphorylation of signal transducer and activator of transcription 5 (STAT5) by activating Janus kinases (JAKs). Furthermore, activated STAT5 binds to the FOXP3 promoter and CNS2, promoting its expression (120–122). On the contrary, when IL-2 signal transduction was deficient, FOXP3 expression stability in Treg cells was lost (123). Interestingly, FOXP3 and other transcription factors jointly inhibit the expression of IL-2 in Treg cells, making it highly reliant on IL-2 produced by activated non-Treg cells, forming feedback control on the expansion of non-Treg cells (124).

Treg cells secrete inhibitory cytokines IL-10, TGF-β, and IL-35. Among them, IL-35 can produce regenerative feedback in Treg cell response by inducing the activation and differentiation of IL-35 producing Treg cells, termed iTR35 (74). Therefore, the significant benefits of IL-35 based therapy lie in the direct inhibition of IL-35 and the synergetic amplification of iTR35 immunosuppression (125).

Furthermore, IL-33, a member of the IL-1 cytokine family, has attracted attention as an important Treg cell immunomodulator recently (126). IL-1 receptor-like 1 (ST2) is considered as the only receptor of IL-33. IL-33 could support the expansion of ST2^+^Foxp3^+^ Treg cells (127), increasing the secretion of inhibitory cytokines IL-10 and TGF-β1 in ST2^+^Foxp3^+^ Treg cells and promoting their suppressive function in head and neck squamous cell carcinoma (HNSCC). ST2 antibody made the opposite effect, which suggested that ST2 may be a potential target for immunotherapy of HNSCC in the future (128). However, IL-33 is also a pro-inflammatory cytokine. In a mouse periodontitis model, systemic administration of IL-33 exacerbated bone loss in a RANKL dependent manner (129). Therefore, given the different roles of IL-33 in different diseases, a deeper understanding of IL-33 action mode in the future will be more targeted at the IL-33-ST2 signal to treat human diseases (130).

### PI3K/Akt/mTOR Signaling Pathway

The PI3K/Akt/mTOR pathway is involved in many biological processes such as survival, proliferation, growth, apoptosis, and glucose metabolism (131). In Treg cells, the activation of PI3K/Akt/mTOR pathway by inflammatory Toll-like receptor (TLR) or T cell receptor (TCR) signals expand Treg cell amplification (132) but reduces FOXP3 expression (133). FOXP3 inhibits Akt phosphorylation and blunts PI3K/Akt/mTOR signal transduction, by which FOXP3 gives a negative feedback regulation and results in enhancing the suppressive function of Treg cells (134). On the contrary, phosphatase and tensin homologues (PTEN), a negative regulator of PI3K, is able to stabilize Treg cells in tumors (135). Moreover, the administration of PI3K-Akt pathway inhibitors in CT26 (a mouse colon carcinoma cell line) mouse models showed a significant therapeutic antitumor effect associated with a selective reduction in Treg cells activation and proliferation with no effect on conventional T cells. It was also demonstrated that PI3K-Akt pathway inhibitors could enhance the antitumor immune responses of the antitumor vaccine by inhibiting Treg cell proliferation (136). Interestingly, mTOR inhibition by rapamycin has been shown to support the proliferation and survival of Treg cells, which is opposed to Akt and PI3K (137–139). Specific deletion of the mTOR gene (Rictor) (140) or inhibition of mTOR activity by rapamycin promoted the induction of FOXP3 and maintained the function of Treg cells (141). On the other hand, the anti-tumor effect of PI3K, Akt, and mTOR inhibitors can directly present as the inhibition of tumor cell proliferation and angiogenesis, as well as the survival enhancement of CD8^+^ T cells (142). Taken together, these data suggested the complicated regulatory mechanisms of the PI3K/Akt/mTOR pathway in Treg cells. Combination of multiple PI3K/Akt/mTOR pathway inhibitors, targeting different steps, may suppress both proliferation and function of Treg cells and achieve better anti-tumor effects.

### Methylation and Post-Translational Modifications

DNA methylation has long been considered as one of the important epigenetic modifications that regulate gene expression but not changing the DNA sequence (143). FOXP3 expression is also regulated by DNA methylation (144). Campos et al. evaluated DNA methylation patterns of 22 gene promoters involved in immune regulation of periapical lesions. The methylation level of the FOXP3 gene promoter was the highest in periapical granulomas and apical cysts, negatively correlated with the expression of FOXP3 mRNA. In addition, active periapical lesions showed higher levels of FOXP3 methylation than inactive periapical lesions. Therefore, the dynamic changes of FOXP3 methylation level at different stages of periapical lesions may regulate Treg cells as a master switch, affecting the process and outcomes of periapical lesions (145).

As for cancer, thymically derived natural Treg cells were suggested as the primary type of Treg cells in tumor tissues and showed a conserved demethylated region in the first intron of the FOXP3 gene. This Treg-specific demethylated region is required for the long-term maintenance of FOXP3 expression (146, 147), which may mediate by the superfluous STAT5 and TET2 in tumor-infiltrating Treg cells (148). Therefore, Treg-specific demethylated region (TSDR) based supportive therapy may provide a novel strategy for anti-tumor immunity treatment *via* reducing intratumoral Treg cells infiltration or weakening its inhibitory function.

Besides epigenetic modification, FOXP3 function is also positively affected by a posttranslational mechanism: FOXP3 arginine methylations. The inhibition of type I protein arginine methyltransferases (PRMTs) is reported to interfere with arginine methylation of FOXP3 and damage the inhibitory function of Treg cells; while up-regulating PRMT1 could prevent Treg cells from tilting to Th1-like cell phenotype (149). PRMTs have been proved upregulated in several tumors, which indicates a poor prognosis (150). Pharmacological ablation of PRMTs is showing a promising prospect of tumor therapy by inhibiting the function of Treg cells (151). At present, the clinical research of various specific PRMTs inhibitors is in full swing and following widely interest.

In addition, other post-translational modifications are also involved in the regulation of Foxp3 functions, such as acetylation (152). Acetylation enhances both stability and activity of FOXP3 (152). Histone acetyltransferases (HTAs) and histone deacetylases (HDACs) have been widely reported to coordinate the differentiation, function, and stability of Treg cells (153). CBP and p300 are the members of the HTAs family. Their combined deletion leads to fatal autoimmunity in mice at 3 to 4 weeks (154). Interestingly, the selective deletion of p300 damages the inhibitory function of Treg cells and enhances anti-tumor immunity without autoimmune deficiency (155). By contrast, HDAC inhibitor therapy usually increases peripheral Treg cells and enhances Treg suppressive function, upregulates acetylation of FOXP3, and related markers like GITR and CTLA-4 (156).

So far, diverse regulatory mechanisms of Treg cell recruitment, proliferation, and function have been reported in oral diseases. Harnessing these mechanisms may help to treat oral diseases (**Table 1**). However, understanding these mechanisms needs to be improved.

**Table 1:** Regulatory mechanisms of Treg cells recruitment, proliferation, and function in oral diseases.

Regulatory mechanisms	Defects or treatments	Effects on Treg cells	Oral diseases	References
CCL22-CCR4 axis	CCR4KO mice; Intraperitoneal injection of anti-CCL22 antibodies	Treg migration impairment	Aggravation of apical periodontitis and periodontitis	(58, 116)
	CCL22-releasing PLGA microparticles	Treg migration promotion	Remission of apical periodontitis and periodontitis	
	CCL22 gene silencing	Treg migration impairment	Impaired oral tumorigenesis	(117)
	CCL22 overexpression	Treg migration promotion	Promoted oral tumorigenesis	
IL-2-JAKs-STAT5 signaling pathway	IL-2KO mice; JAKsKO mice; STAT5a/b double KO mice	Reduction of Treg frequency	Unknown	(121)
	Transient activation of STAT5 in IL-2-deficient mice	Increasing Treg number		
IL-35	Intragingival injections of IL-35	Increasing induction of iTr35 cells	Inhibition of periodontitis progress	(74)
IL-33	IL-33 overexpression	Expansion of Treg population and function	Poor prognoses of HNSCC	(128)
	Anti-ST2 mAb	Inhibition of Treg number and function	Promotion of effector T cell proliferation	
PI3K/Akt/mTOR signaling	Targeting PI3K and Akt with specific inhibitors	Inhibition of Treg proliferation	Enhancement of the antitumor immune response	(136)
	Rapamycin (mTOR inhibitors)	Expansion of Treg	Inhibition of effector T cell function	(137)
FOXP3 gene methylation	Hypomethylation	Promotion FOXP3 expression; Increase of Treg infiltration	Inactive apical periodontitis; Promoted tumorigenesis	(145, 147)
FOXP3 arginine methylation	Targeting PRMTs	Inhibition Treg function	Enhancement of the antitumor immune response	(149)
FOXP3 histone acetylation	Selective deletion or pharmacologic inhibition of p300	Inhibition Treg function	Enhancement of the antitumor immune response	(155)

## Concluding Remarks

Oral diseases, as one of the most common public health problems worldwide, are closely associated with immune disorders. When patients suffer from autoimmune diseases such as rheumatoid arthritis and systemic lupus erythematosus, oral manifestations such as chronic periodontitis, oral lupus erythematosus, and Sjogren’s syndrome are also common (157, 158). However, when the body’s immune function defect, whether primary or acquired, it is often accompanied by necrotizing ulcerative periodontitis, oral candidiasis, and the risk of tumor is significantly increased (159). Treg cells play important roles in maintaining immune homeostasis and self-tolerance in oral tissues. They play protective roles in inhibiting the course of inflammatory diseases such as apical periodontitis and periodontitis but accelerate the deterioration of precancerous lesions in oral mucosa and HNSCC. Therefore, Treg is a promising immunotherapeutic target of oral diseases. So far, the regulatory mechanisms of Treg distribution, stability, and function remain largely unclear. Further researches are required to explore these mechanisms and help to design Treg-based therapeutic strategies for the treatment of oral diseases.

## Author Contributions

YZ, JG, and RJ wrote the manuscript. All authors contributed to the article and approved the submitted version.

## Funding

This work was supported by grant from Health Commission of Hubei Province Scientific Research Project (grant no. WJ2019Z014), and Key Research and Development Program of Hubei Province (grant no. 2020BCB046).

## Conflict of Interest

The authors declare that the research was conducted in the absence of any commercial or financial relationships that could be construed as a potential conflict of interest.
